# AI-Enabled, Text-Based Health Coaching and Navigation for Employees to Support Health Outcomes: Pre-Post Observational Study

**DOI:** 10.2196/64553

**Published:** 2025-09-30

**Authors:** Paula Wilbourne, Susan Mirch-Kretschmann, Denise Walker, Michael Varghese, Roberto Arnetoli

**Affiliations:** 1 Sibly, Inc, 400 Concar Dr., San Mateo, CA, 94402, United States, +1 801 855 6493; 2 RecoveryWorksAI, Pacifica, CA, United States; 3 VA Palo Alto Health Care, U.S. Department of Veterans Affairs, Palo Alto, CA, United States; 4 School of Social Work, University of Washington, Seattle, WA, United States

**Keywords:** digital mental health, machine learning, health coaching, behavioral, artificial intelligence, quality of life, interviewing, proof of concept, scalability, access

## Abstract

**Background:**

Limited, timely access to quality mental health treatment harms well-being and quality of life while costing individuals and organizations millions in increased medical spending and reduced productivity. Too few qualified professionals, inconsistent quality, and stigma thwart traditional solutions, creating the need for scalable, science-based solutions.

**Objective:**

This report provides an overview of a novel digital health coaching service that consists of artificial intelligence (AI)–assisted, human-delivered, text-based health coaching. This report provides data evaluating the efficacy of this service for delivering mental health support, improving well-being, and enhancing workplace productivity.

**Methods:**

This observational study analyzed operational and self-reported health data from employees of subscribing organizations who used Sibly’s digital health coaching service. Data included response times, changes in expressed member sentiment, conversation topics, and adherence to motivational interviewing. A subset of members (n=38) provided pre-post self-reported assessment measures of distress, unhealthy days, and presenteeism, having engaged in at least 4 coaching conversations over a minimum of 14 days. Sentiment was evaluated using a natural language processing tool.

**Results:**

Sibly provided quick access to interactive human coaching, with a median response time of 132 seconds. Sentiment analysis showed that 57% (878/1540) of conversations increased in positive emotions. The coaches maintained strong fidelity to the techniques of motivational interviewing, with adherence exceeding 90% (387/430). The proportion of users reporting severe distress declined from 33.3% (10/30) at baseline to 6.7% (2/30) at follow-up, representing a 79% relative reduction (*P*<.001). Participants also reported a reduction in the number of unhealthy days per month, decreasing from 19.57 to 15.87 per month (*P*=.02). Self-reported productivity improved by 18% during the study period (*P*<.001). Additionally, 61% (47/77) of users who received referrals to additional employer-sponsored benefits engaged with those resources, demonstrating effective care navigation to relevant support services.

**Conclusions:**

This report provides an overview of novel mental health support and navigation services that use AI-enabled, text-based health coaching and care navigation. Data suggest that the services provide effective, scalable mental health support in workplace settings. The platform helped reduce distress, improve well-being, and boost productivity by offering immediate access to trained coaches and personalized guidance. These results are consistent with existing research on digital mental health services. They highlight the potential of AI-assisted coaching to improve access to care. Future research should include larger, diverse populations and more rigorous randomized controlled trials. This formative report provides data that describes and demonstrates a proof of concept for an innovative technology-enabled service that addresses the problems of scalability, access, quality, and stigma that challenge the provision of traditional mental health services.

## Introduction

### Mental Health Service Need

Untreated mental health problems are costly, having been linked to physical health problems, lower treatment adherence, and shorter lifespans [1,2]. Even before the pandemic, rates of emergency room visits for people with mental health diagnoses and comorbidities increased nationwide [3]. Finally, employees distressed at work impact culture, lower productivity of those around them, and burden human resource services [4].

In the years since the pandemic, the growing demand for mental health treatment has collided with the longstanding limitations in our capacity to provide it. Mental health provider shortages predated the pandemic and have worsened, with wait times averaging 5‐6 weeks [5-7]. Unfortunately, patients do not respond well to treatment delays with longer wait times, resulting in greater no-show rates [8]. The pandemic has worsened mental health, straining an already burdened system. Only 20% of adults who needed emotional support received help from a mental health professional [8]. Issues such as location, timing, and provider capacity (45%); costs (39%); and not knowing how to find help (21%) prevented access to care. Other barriers included privacy concerns, not thinking their problems are serious enough, concerns about stigma, or other people finding out [9]. Traditional entry to care is designed for more severe conditions, while 76‐90% of distressed populations do not want or need higher levels of care [10,11]. Evaluations of stepped-care models find that only about 20% of those seeking help needed medical treatment [11]. This mismatch wastes resources, can be off-putting to treatment seekers, and creates inefficiencies for efficacious treatment.

A large body of research identifies effective, efficient tools that hold promise in the face of the current mental health crisis. Despite strong evidence, traditional mental health and medical services have not taken robust advantage of this research. We will briefly review stepped care, the use of trained coaches and paraprofessional staff, digital interventions, text-based interventions, empirically based brief interventions, and measurement-based interventions as a foundation for the program that follows.

Quality often refers to evidence-based practices and the outcomes associated with those interventions [12]. Effective solutions to the mental health crisis must measure outcomes and ensure fidelity to empirically supported models to deliver the promised track record of efficacy. Using text-based interventions and real-time natural language processing (NLP) provides an opportunity to ensure that the services deliver quality in the form of adherence to evidence-based protocols. To be successful, quality, accessibility, and efficacy must be accomplished with fewer staff members, at a low cost, and promptly to meaningfully address the problems of existing solutions.

Stepped-care models provide lower levels of high-quality assistance, improve outcomes, and expand the capacity and accessibility of current mental health resources. Timely assistance (eg, self-help, digital tools, and coaches) can lead to lasting improvements [13-22]. Those who have to wait for assistance often continue to experience symptoms, get worse, and may never reach the same degree of improvement as those who get assistance quickly [13-18]. The advantages of self-help, digital resources, and nonmedical assistance, as initial steps in a stepped-care model, have been demonstrated in multiple studies of depression, substance use, anxiety, insomnia, and overall mental health symptoms [14-17,21-29]. Early initial steps of care prevent more severe mental health problems [13,14,23,30-33].

Trained coaches and nonprofessional staff provide another science-based option to address barriers to effective mental health treatment. Well-trained and supervised unlicensed staff can achieve similar outcomes as licensed professionals [34-36] when delivering well-specified interventions like motivational interviewing and cognitive-behavioral interventions [32,35-38]. Trained coaches and unlicensed staff have demonstrated both treatment fidelity and efficacy in the treatment of anxiety and depression [39] and in facilitating health behavior change [34,36-38]. The World Health Organization concluded that specialized staff are not required to deliver mental health intervention [40].

Fortunately, flexible options for delivering assistance, such as text and asynchronous support, go beyond traditional face-to-face or even video telehealth. SMS text messaging reduces the barriers to mental health treatment noted above, including access, location, timing, stigma, and privacy [41,42]. Digital content materials and SMS text messaging are effective tools [16,18,24,42,43]. Digital text messages written by trained coaches expand accessibility and lower the cost of mental health services with outcomes comparable to telephone coaching [39,42-49]. Text has become a ubiquitous form of communication and is the preferred method of communication for adults under 50 years of age. Over two-thirds of adults value SMS text messages from health care providers, and more than 41% are “constantly online” [50-52].

The potential of artificial intelligence (AI) in mental health care represents a revolutionary shift in managing mental health problems. Interactive and supportive chatbots make use of large language models, NLP, and machine learning (ML) to provide real-time interaction, creating a safe environment for users and offering immediate coping strategies, which may have the potential to address the shortage of mental health treatment resources [53,54]. NLP detects sentiment and language features linked to mental health symptoms. NLP can also evaluate the quality of care and adherence to evidence-based interventions demonstrated in clinical interactions. In general, using digital interventions and AI allows for real-time optimization of mental health interventions [55,56].

One aspect of NLP, sentiment analysis, has been applied to mental health content in the published literature. Sentiment analysis measures the attitudes, sentiments, evaluations, and emotions of a speaker or writer based on the computational treatment of subjectivity in a text [55]. It is generally performed using rule-based tools and lexicons to calculate the semantic orientation of words and phrases in a text. A sentiment score between −1 and +1 indicates generally negative or positive sentiment, respectively (0=neutral sentiment). Sentiment analysis related to mental health concerns has been conducted on large text datasets, including those drawn from social media, electronic health records, narrative writing, and less frequently on smaller samples of the content of interviews or narrative writing samples [57-59].

### Sibly: An Innovative Approach to Health Behavior Change

Sibly provides 24/7/365 digital coaching via text with trained human coaches to organizations that pay a fee to provide the service to their members. The platform integrates with the health and wellness resources of these organizations, allowing health coaches to navigate members to timely, effective referrals as needed during coaching. The platform gathers member and coach data to enhance service delivery.

Members maintain anonymity through display names, creating a secure channel for receiving coaching and educational materials related to health behaviors, life challenges, and mental health. The mobile-friendly platform eliminates barriers of location, waitlists, stigma, and timing, offering on-demand support.

Members use a messaging app and are assigned to a small team of health coaches, who respond collectively as “Sibly.” Trained health coaches, using evidence-based practices such as motivational interviewing, mindfulness, coping skills training, and cognitive-behavioral tools, help members set goals and take action. In addition, Sibly’s health coaches guide members to use self-help materials, sponsored benefits, and community resources that are recommended based on each member’s individual needs.

Members receive unlimited coaching and progress reports. Baseline and follow-up well-being assessments are conducted. Personally identifiable information (ie, name, address, phone number, and date of birth) is collected and used only for emergencies (danger to self, others, or vulnerable persons) by PhD-level experts who respond in less than 30 minutes.

Protocols on the scope and development of text-based coaching proposed 5 domains: selection and training of coaches, specific coaching techniques, how to structure communication with those being coached, monitoring adherence to guidelines, and quality of coaching [39]. Sibly uses strategic recruitment and in-house, science-based coach training to address provider shortages and scale services. Successful applicants must have a bachelor’s degree and are behaviorally evaluated for empathy, coachability, and professionalism. Qualified candidates are invited to participate in a paid training program.

Training consists of 240 hours of competency-based instruction in listening skills, motivational interviewing, cognitive-behavioral tools, mindfulness, and crisis response, grounding the service in rigorous, science-based interventions. New health coaches demonstrate competence during observed training cases. To date, 98 health coaches have entered training, with 94.9% (93/98) successfully completing it. Training each coach costs approximately US $4800. Approximately 40% of these costs are offset by the services they provide to members during training.

Quality assurance ensures health coaches retain and improve their skills. Sibly health coaches participate in quarterly continuing education classes and receive monthly feedback on their coaching skills from expert trainers using the adapted Motivational Interviewing Treatment Integrity (MITI) coding system [60]. A proprietary tool using ML and AI detects adherence to training skills and provides real-time feedback. Ninety-five percent of coded quality assurance conversations meet competency guidelines similar to those specified by the MITI. Health coaches not meeting competency standards participate in weekly conversation reviews until they improve. Those unable to improve are disqualified from continuing their work as health coaches.

AI and ML play a crucial role in supporting Sibly’s human health coaches and structuring communication with members. AI suggests optimal next steps, identifies service improvement opportunities, enhances engagement, and improves the member experience. ML provides the health coaches with insights in real time that might not occur to the coach, or that may be based on previous conversations. Sibly’s ML tools reliably detect 40 specific topics, measure sentiment fluctuations, identify the start and end of a text conversation, and suggest optimal next steps to the health coaches. The topic models were designed to reduce bias by accurately representing topics related to race, gender, and sexual orientation that are less frequent in the population and typically overlooked in models that are not specifically trained to detect them.

The platform provides health coaches with digital notifications or “nudges.” Nudges assist the health coaches by highlighting information and recommending the next steps. Nudges personalize recommendations based on member priorities and goals. For example, if a member is interested in weight loss, nudges suggest relevant content and employer-sponsored benefits related to weight loss. If a member is concerned about their mood, nudges can suggest screening measures, self-help materials, and employer-sponsored mental health benefits related to this member’s concern. The health coaches refine AI nudges through real-time feedback to the model.

The text-based communication platform allows for rigorous quality assurance and data analytics. AI and ML offer real-time feedback, reducing skill drift that can occur with episodic training. Through AI-assisted health coaching, Sibly is designed to provide personalized, high-quality, human-led coaching, resource navigation, and crisis response that is science-based, data-informed, efficient, consistent across the health coaches and, therefore, more scalable.

The current report includes observational data that describes the structure, service level, and impact of a novel digital health platform. We report on a subset of members who provided pre-post self-report questionnaires on mental and physical well-being.

## Methods

### Sample

We report on two data samples. First, over a 5-month period, all new members were asked to complete a baseline assessment after completion of their first coaching conversation. After 2 weeks and completion of the fourth coaching conversation, these members were asked to complete a follow-up assessment. Thirty-eight members who completed a baseline assessment went on to complete 3 additional coaching conversations and were asked to complete a follow-up questionnaire. Of the 38 eligible participants, 30 (78.9%) agreed to complete the follow-up.

All 38 members meeting these eligibility criteria were detected using ML-enabled tools that prompted the coach to send the follow-up survey within 15 minutes of the end of the fourth coaching conversation. On average, the conversations of the original 38 members completing the baseline assessment involved the exchange of 25 messages, 11 of which were from the member. There were no significant differences in the length or timing of conversations between those completing a follow-up assessment and those who did not.

Sample selection reflects a combination of both theoretical and practical considerations. Previous product analyses suggest that members are more likely to complete a questionnaire after exchanging 10 messages with a coach; therefore, the baseline symptom assessment was sent after the end of the first conversation. The follow-up questionnaire was sent after members completed their fourth conversation to allow credible exposure to the service in the eyes of our customers. We limited the window of time to 14 days to reduce the chances that members might be lost to follow-up.

Second, we report data on our larger sample of operational data for the entire population of members using the service. The number of participants available for analysis is specified in each analysis below. All data available were included. No participants were selectively omitted from these analyses.

### Ethical Considerations

During registration, users confirmed that they were at least 18 years old and consented to the use of their deidentified aggregate data. This study received a retrospective exemption from the institutional review board of the University of Washington Human Subjects Division (STUDY00023309) and was not considered human subjects research due to the observational and deidentified nature of the data collected.

All data management and analysis adhered to our terms of service and privacy policies, to which members agreed at enrollment. Messaging, operational, and survey data analyzed for ML were anonymous and processed in a separate system from member-identifying information. Data were reported only in aggregate and encrypted for privacy. Three data sources are reported here, including pre-post self-reported health data, operational data, and an ML analysis of the sentiment measured in participant conversations.

### Quantitative Variables and Operational Data

Response time was measured as the seconds between a member’s message and the coach’s reply. Sequential member messages were counted as one.

The digital platform and phone app allowed us to measure, analyze, and optimize a number of product dimensions: response rates, time of day, sentiment, coaches per conversation, topics, and adherence to motivational interviewing. These data provide insight into the performance of the product and ways in which members used the service, which will be reported below.

### Self-Reported Health-Related Questionnaire Data

Self-report measures were administered within the app. Pre-post data were then analyzed using a paired samples *t* test to determine the significance of changes made by participants using the service. Self-reported measures included the Lam Employment Absence and Productivity Scale (LEAPS) [61,62], health-related quality of life as measured by the Center for Disease Control and Prevention’s Healthy Days [63], a 2-item measure of mental anguish and distress [64,65], and a single item assessing the member’s evaluation of the change in mental well-being as a result of their use of the service.

The LEAPS is a 10-item scale with documented internal consistency and external validity. It took 3‐5 minutes to complete and was administered as a measure of the degree to which a participant’s work performance was impacted by their mental health symptoms [60,61]. With the author’s permission, distress was measured using 2 items assessing mental anguish that was first published in 2013 and later developed into a 10-item scale in 2019 [62,63]. The questions and response options are as follows: How much are you suffering emotionally (mental anguish, not pain, or discomfort in your body)? 1=absent; 2=very mild or occasionally; 3=mild, comes in moments and goes away; 4=moderate, steady and in specific moments; 5=marked, hurts all the time and does not get better; 6=severe, unbearable; 7=extreme, feels like you want to die; and 8=skip. A face-valid question assessing a member’s change in mental well-being attributable to Sibly—“To what degree has your mental well-being changed as a result of working with Sibly?”—with response options that included 1=much better, at least 50% better, or improved on most days; 2=better, at least 25% better, or improved 2‐3 days per week; 3=a little better, some improvement, or better on 1 day per week; 4=no change; 5=a little worse, somewhat worse on at least 1 day per week; 6=worse, at least 25% worse, or worse 2‐3 days per week; 7=much worse, at least 50% worse, or worse on most days.

### Sentiment Analysis

To assess the impact of coaching on sentiment, we analyzed the change in sentiment or sentiment shift. First, within a conversation, sentiment shift was defined by the change in sentiment from the first 5 messages of the conversation to the last 5 messages of the conversation. Second, sentiment shift was measured from the start of each member’s first conversation to the end of the conversation, occurring immediately prior to their completion of the follow-up questionnaire. We used VADER (Valence Aware Dictionary and Sentiment Reasoner), a sentiment analysis tool designed to process informal text, including slang, emojis, and punctuation. VADER is part of the Natural Language Toolkit [66,67], a leading suite of Python libraries and programs for NLP. A sample of our first 1512 conversations with B2B (business-to-business or participants whose membership was paid for through an employer) members was analyzed to look at the number of coaches per conversation. These reflect the total number of conversations with employee members that had taken place at the time of this analysis. No conversations or members were omitted. A chi-square test was used to look at the relationship between the length of the conversations and the sentiment of the conversation.

### Coaches per Conversation

We analyzed 1549 B2B conversations to look at the number of coaches per conversation. These reflect the total number of conversations with employee members that had taken place at the time of this analysis. No conversations or members were omitted. A chi-square test was used to look at the relationship between the number of coaches per conversation (1-7) and members’ experience of those conversations as reflected by the percentage of conversations with a positive sentiment shift.

### Topics

We used an unsupervised topic modeling technique, Latent Dirichlet Allocation (LDA), to identify conversation topics. LDA is a generative statistical model used in NLP and ML to identify thematic structures within large text datasets. Overall, we used 3 trained health coaches as coders to label the topics detected by the models. We then asked the coders to rate the degree to which the proposed models captured the topics discussed in 100 randomly selected conversations. The output and evaluation of this process are reported below.

## Results

### Response Time

Coaches responded to member messages with a median response time of 132 seconds and an average of 197 seconds.

### Optimizing Questionnaire Response Rates

Before December 2021, approximately 29% (2795/9565) of our requests to members to complete surveys were acted on. To improve this response rate, we examined optimal points in the member relationship (number of messages sent) and the timing in the conversation at which members were most likely to complete a questionnaire or survey (within 15 minutes of the end of a conversation). Optimizing survey administration along these dimensions increased our response rate to 48.6% (2041/4201; *χ*^2^_1_=485.4, n=13,766, *P*<.001).

### Time of Day and Work Hours

**Figure 1.**
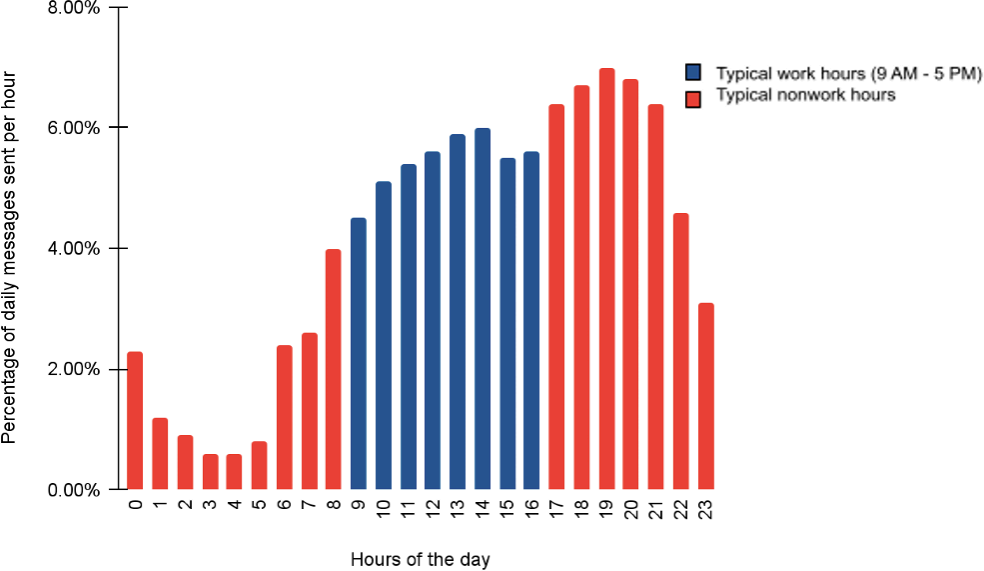
Distribution of employee messages by time of day, 2021‐2023.

Fifty-six percent (8938/15,960) of our member messages were sent outside of the hours of 9 AM to 5 PM, suggesting that most employees are accessing assistance without disruption to their workday and outside the hours that most mental health services would be available to them (see Figure 1). During work hours, members take twice as long to respond to coaches as they do outside of work hours, suggesting the ability to fit coaching conversations amid other demands on their time.

### Sentiment

Over each member’s relationship with Sibly, the slope of the sentiment line between the beginning of the first conversation and the end of the conversation occurring before the follow-up assessment was positive for a majority of the participants. Twenty-one (70%) of the sample of 30 members completing pre-post measures demonstrated a positive shift in sentiment, while 9 demonstrated a negative shift in sentiment. Within individual conversations, an average of 57% (878/1540) demonstrated a positive sentiment shift. This information provided insight into the impact of coaching conversations on member sentiment that occurred during the member conversations and over the initial coaching conversations. In our larger member population, there was a positive correlation between the length of member conversations with sentiment shift suggesting that the longer a member talks with a coach, the better they feel or that the better a person is feeling, the longer they are likely to continue talking to Sibly (*χ*^2^_5_=58.01, n=1512, *P*<.001).

### Coaches per Conversation

In a larger sample of 1549 member conversations, 70% (1084/1549) of conversations were completed by a single coach, 23% (356/1549) of conversations included responses from 2 coaches, while 7% (108/1549) of conversations included 3 to 7 coaches. Sentiment analysis did not detect a decrease in sentiment associated with conversations that switched between one coach and another. In fact, conversations with more than one coach were longer and demonstrated a greater improvement in sentiment than those in which a member spoke to fewer health coaches during the conversation (*χ*^2^_3_=17.701, n=1549, *P*<.001). Generally, members are not aware that they have spoken to more than one coach during a single conversation. However, in <2% (23/1549) of conversations, members express concern or dissatisfaction about working with more than one coach or the perceived change in coach during a conversation.

### Topics

Using an unsupervised topic modeling technique, we developed and evaluated 7 possible models with 30, 40, or 50 topic clusters. Topics were labeled, and labels were validated by 3 independent coders. We then asked the coders to rate the fit of these models on a 5-point Likert scale for 100 randomly selected conversations. Models A-F were rated as “best reflecting the conversations” as follows: A (45/100, 45%), B (21/100, 21%), C (17/100, 17%), D (10/100, 10%), E (10/100, 10%), F (2/100, 2%), and G (1/100, 1%) of the time. Model A, with 40 topics identified, was selected as the one best reflecting the conversations 45% (45/100) of the time, with the topics detected in model A evaluated as fair to very good 84% (84/100) of the time.

Applying the selected model, we found that members came to the first conversation with an average of 5.6 topics, where over 76% (12,129/15,960) presented with at least 3 interconnected topics of conversation. Thirty-one topics of conversation were detected across all member conversations in this sample. Negative emotions, work, family, relationships, and mental health were the most common topics of conversation, occurring in more than 20% of conversations. Self-organization, self-improvement, employer-sponsored benefits, health behaviors, and love were discussed in more than 10% of conversations. Twenty-one additional topics occurred in fewer than 10% of the conversations. See Table 1 for all topics.

**Table 1.** Machine learning–detected topics from member-initiated messages (n=15,960).

Topic	Values, n (%)
Negative emotions	5108 (32.0)
Work	5152 (32.3)
Family	4739 (29.7)
Relationship	3783 (23.7)
Mental health	3377 (21.2)
Self-organization	2626 (16.5)
Self-improvement	2472 (15.5)
Discussing benefits	2457 (15.4)
Health behaviors	2417 (15.1)
Love	2319 (14.5)
Sleep	1385 (8.7)
Emotions: hope	1252 (7.8)
Living situation	1227 (7.7)
Hobbies	1188 (7.4)
Coping strategies	1126 (7.1)
Divorce	1115 (7.0)
Finance	1115 (7.0)
Medical problem	1090 (6.8)
Education	1050 (6.6)
COVID	1025 (6.4)
Goals focusing	875 (5.5)
Religion	666 (4.2)
Happy fun	610 (3.8)
Relaxation	548 (3.4)
Racism	337 (2.1)
Legal	272 (1.7)
LGBTQ+	265 (1.7)
Friendship	257 (1.6)
Disability	252 (1.6)
Politics	113 (0.7)
Gender	40 (0.3)

### Evidence-Based Practice Adherence

During the period in which data were collected, 2 conversations per coach per month were coded for adherence to motivational interviewing using the Sibly quality assurance manual, which was based on the MITI scale [60]. This resulted in 430 coded conversations, of which 387 (90%) were determined to be adherent to motivational interviewing.

### Distress

Participants were asked to rate their levels falling into categories of “1=mild or none” for those indicating “absent or occasionally,” “2=moderate” for those indicating “comes and goes or steady,” and “3=severe” for those indicating that it “hurts all the time, unbearable, and feels like they want to die.” These groupings were chosen to aid in the interpretability when presenting to nonclinical audiences. These were referred to triage with PhD, per previously mentioned procedure, earning scores 1‐3, respectively, at both baseline and follow-up. At baseline, participants reported an average distress level of 2.27, with 33.3% (10/30) reporting severe distress. After using the service, average distress decreased to 1.87, with an 80% decrease in those reporting severe distress and a 200% increase in those reporting mild or no distress. A paired samples 2-tailed *t* test found a significant improvement in average distress levels, *t*_29_=−3.89 (*P*<.001), and a medium effect size *d*=0.71.

### Unhealthy Days

Participants were queried as to the number of unhealthy days in the past month attributable to mental health and then physical health. These numbers were added together up to a maximum of 30 days, consistent with the scoring recommendations for this measure by the Centers for Disease Control. Participants also reported an average of 19.57 unhealthy days per month at baseline, with 60% (18/30) experiencing poor health (16+ unhealthy days/month). At follow-up, unhealthy days decreased to 15.87 (*P*=.02, *d*=0.42), and the proportion of members in poor health dropped to 36.7% (11/30).

### Productivity or Presenteeism

Analyzing data from the LEAPS questionnaire found that at baseline assessment, participants scored an 8.5, which falls in the mild range for productivity impairment due to mental health symptoms at work. At follow-up, participants reported ~18% (1.5/8.5) decrease with a mean score of 7. A paired samples 2-tailed *t* test found a significant improvement in productivity, *t*_29_=–3.99 (*P*<.001), and a medium effect size *d*=0.71.

### Benefit Referral and Engagement

During the evaluation period, the health coaches recommended 18 benefit programs to 14 participants. Members engaged with 11 (61.1%) of the benefit services recommended to them. Additionally, among a sample of 77 participants who were referred to their EAP program, 46 of these participants enrolled in that benefit program. For comparison, a third-party comparison of Sibly to more traditional benefit navigators found that Sibly’s referral success rate was 3 times higher than that of more traditional navigation services.

## Discussion

This paper describes a novel AI-enabled, text-based health coaching platform that supports mental health via immediate, accessible assistance from live human coaches. Analysis found that the health coaches respond to members in less than 3 minutes. Objective quality assurance ratings found that more than 90% (387/430) of conversations were adherent to motivational interviewing and that 56% (8938/15,960) of member messages were sent outside of traditional clinical service hours of 9 AM to 5 PM. While 70% (1084/1549) of member conversations were conducted by a single coach, sentiment did not decline when multiple coaches participated; instead, conversations with multiple coaches were longer, with a greater improvement in sentiment. Over time, a majority of members showed an increase in positive sentiment between their first and last conversations and within the majority of individual conversations. The most common topics of conversation included negative emotions, work, family, relationships, and mental health. Other common topics included self-organization, self-improvement, employer benefits, health behaviors, and love. A subset of members providing pre-post self-report data reported an 80% decrease in severe distress, a 19% decrease in unhealthy days, and an 18% increase in productivity. Sixty-one percent of the time that a health coach referred a member to an additional employer-sponsored benefit, the referral was successful. The results demonstrate the potential of AI-supported text coaching as an efficient, scalable, and effective workplace mental health solution.

There are important implications for the key findings that highlight the speed of access, rapid response times, consistent use of empirically based tools, and improvements in distress, productivity, and engagement with employer-sponsored benefits. The analyses presented indicate that the service increases accessibility for individuals who may not want or cannot schedule therapy, reduces delays in care, and holds a credible promise to supplement traditional services, especially in those who need immediate support.

The service uses a one-to-many model, a novel and efficient way to scale the human coaching relationship. Each member is assigned to a small team of coaches who are selected for empathy and are trained in empirically based tools, allowing the coaches to speak with a similar voice and deliver a consistent intervention. The analysis reported shows that coach transitions within a single conversation do not impact member sentiment. This finding supports the credibility of the one-to-many model, where each member is assigned to a small team of coaches who respond as a single entity. Sibly’s digital platform and AI support enable a seamless collaboration among providers, which is difficult to achieve in traditional care.

Members most frequently discussed topics of negative emotion, work, family, relationships, and mental health and aligned with previous findings that work-related stress, personal relationships, and emotional distress significantly impact mental health challenges. The fact that work stress ranked second underscores the importance of workplace mental health solutions, and these findings suggest that text-based coaching may improve mental well-being and workplace functioning. Previous research has found that mental health interventions enhance productivity and reduce absenteeism at work [68].

These findings are consistent with prior research on digital mental health interventions, which have been shown to improve access to care and support well-being in a scalable way [69]. Stepped-care models suggest that lower-intensity interventions, such as text-based coaching, can be highly effective for individuals who do not require or want more traditional therapy or clinical treatment [60]. By integrating benefit navigation, Sibly addresses a common challenge seen in both mental health support and employer-sponsored benefits—helping individuals connect with appropriate resources when they are needed. ML also played a role in increasing engagement and response rates, helping to optimize interactions in ways that traditional benefit navigation methods cannot do. This suggests that AI-assisted coaching could enhance accessibility and service quality while also supporting employee well-being and workplace productivity.

Despite these encouraging results, this study has several limitations. Because it was observational and lacked a control group, we cannot conclude with certainty that Sibly was the direct cause of the observed improvements. While members reported significant reductions in distress and unhealthy days, external factors could have influenced these outcomes. Additionally, the sample size was relatively small and composed of employees with access to employer-sponsored benefits, meaning the results may not generalize to broader populations. Furthermore, employees self-selected to use the service, which may introduce selection bias, as those who engaged with the platform might differ systematically from those who did not. As a result, the findings may not fully reflect the experiences or needs of the larger employee population. Future research should aim to include larger and more diverse samples, as well as implement randomized controlled trials to establish causality. While sentiment analysis and engagement metrics provide useful insights into user experiences, more qualitative research is needed to understand how members perceive and engage with the platform.

The broader implications of these findings highlight the increasing role of digital health coaching in expanding access to mental health support and reducing the burden on traditional clinical services. AI-enabled platforms like Sibly offer a promising, scalable, and cost-effective solution to the growing need for workplace mental health solutions. Digital services have the potential to shift mental health care from a reactive, appointment-based medical model to a proactive, on-demand system. As AI and NLP continue to advance, these services will likely become more precise, responsive, and personalized to individual users. Additional research and transparent evaluation will be crucial in refining these models and ensuring that digital mental health solutions continue to meet the evolving needs of individuals and organizations alike.

## Abbreviations

AI: artificial intelligence
B2B: business to business
LDA: Latent Dirichlet Allocation
LEAPS: Lam Employment Absence and Productivity Scale
MITI: Motivational Interviewing Treatment Integrity
ML: machine learning
NLP: natural language processing
VADER: Valence Aware Dictionary and Sentiment Reasoner
